# Mapping Research on Early Ethnic‐Racial Awareness Development Among Infants and Toddlers: A Scoping Review

**DOI:** 10.1111/infa.70040

**Published:** 2025-08-08

**Authors:** Anissa L. Eddie, Claire D. Vallotton, Holly Brophy‐Herb, Loria Kim, Carin Graves, Danielle Dalimonte‐Merkling

**Affiliations:** ^1^ Department of Human Development and Family Studies Michigan State University East Lansing Michigan USA; ^2^ Department of Human Development and Family Sciences University of Connecticut Storrs Connecticut USA; ^3^ Michigan State University Libraries East Lansing Michigan USA; ^4^ Michigan State University Extension East Lansing Michigan USA

**Keywords:** anti‐bias, ethnic‐racial awareness, ethnic‐racial socialization, infants, toddlers

## Abstract

The purpose of this study is to expand understanding of the early stages of the lifespan model of ethnic‐racial identity by summarizing and mapping existing research on the development of ethnic‐racial awareness among children from birth to age 3. A scoping review methodology is used to systematically identify and analyze the existing literature on early ethnic‐racial awareness and developmental influences on this awareness among infants and toddlers. The final analysis included 168 unique studies within 105 papers published between January 1990 and March 2023. Findings confirm that infants and toddlers demonstrate a capacity for ethnic‐racial awareness, including phenotypic appearance and language of those similar and different from their own. Findings also demonstrate the need for more research on individual differences in the development of ethnic‐racial awareness, and the influences that account for variation in order to further understand how ethnic‐racial awareness emerges and evolves during the racial‐priming period. Published research on ethnic‐racial awareness among children under 3 years of age primarily includes looking time studies with disproportionate samples of White infants. Findings also indicate an absence of studies examining early ethnic‐racial socialization practices and anti‐bias interventions among caregivers of infants and toddlers. Implications for future research are discussed.

## 1 Introduction

Research that is focused on the developmental period of infancy has been critical to understanding how aspects of human development originate and then continue across the life span. As infants are innately drawn to human faces (Bastianello et al. 2022) and completely dependent on primary caregivers (Hart 2022), preferences for, attention to, and perception of human faces have long been a focus of infancy researchers. One specific line of research that has evolved from such studies is the exploration of how infants respond to faces from different ethnic and racial groups (e.g., Bar‐Haim et al. 2006; Hayden et al. 2012; Katz and Kofkin 1997). Although many studies on this topic have been conducted, there has been no attempt to synthesize this research and put it in the broader context of ethnic‐racial identity (ERI) development. Yet, as with so many aspects of human development across the lifespan, the origins of ERI can be traced to infancy (Williams et al. 2020). ERI has typically been discussed in terms of individual identify formation in relation to understanding and internalizing one’s own ethnic background and racial group membership (Ruck et al. 2021). This level of cognitive processing is not accessible to infants, but the perception and processing of individuals from familiar and unfamiliar groups emerges in infancy and may provide the foundational elements of ERI development (Williams et al. 2020).

### 1.1 The Lifespan Model of Ethnic‐Racial Identity

The lifespan model of ethnic‐racial identity (the lifespan model) offers a way to understand the evolution of ethnic‐racial identity starting with its origin and extending across all developmental periods of the human lifespan (Williams et al. 2020). The impetus for this work was to connect the “…relatively piecemeal approach [that] makes it difficult to chart continuity across developmental periods…” (Williams et al. 2020, 100). The resulting integrated model provides a helpful overarching frame for the construct of ERI. The lifespan model asserts that ERI is “…a process that begins in early infancy and progresses throughout late adulthood” (Williams et al. 2020, 101). The earliest stage of ERI development, referred to as the phase of “ethnic‐racial priming” starts in early infancy and extends to age 3 (Rogers et al. 2020, 105). When considering this initial phase, it is important to situate the research within the connection between ERI and ethnic‐racial socialization (ERS).

The use of the term *ethnic‐racial* is reflective of expansion in the field (Hughes et al. 2006; Markus 2008). Many current scholars have shifted to use of the combined term, instead of racial and ethnic separately, to encapsulate the entirety of the construct (Doucet et al. 2018; Umaña‐Taylor et al. 2014). This combined usage is seen in both ERI and ERS language. While closely connected, ERI and ERS are unique constructs. ERI comprises the ways in which one perceives, describes, and values their personal ethnic‐racial group membership, and how individuals define their sense of self based on the ethnic and racial backgrounds with which they feel affiliated (Umaña‐Taylor et al. 2014). ERS refers to the messages that children receive regarding ethnicity and race (Hughes et al. 2006; Quintana 1998). These messages come from caregivers (e.g., parents, families, and educators), environments (e.g., home, neighborhood, school, geographic region), and media (e.g., books, toys, television, and technology tools; Williams et al. 2020). Through these socializing agents, children learn about common norms, values, and customs that may be related to both their own ERI and that of others (Hughes et al. 2006). ERI and ERS are connected in that the quality, frequency, and content of the ERS messages children receive directly impacts the way in which their ERI takes shape (e.g., Hughes et al. 2023). Taken together, the lifespan model provides a cohesive model that connects ERI development across all developmental periods. However, the lifespan model is also strengthened by in‐depth research within precise developmental periods.

### 1.2 Research From the Ethnic‐Racial Priming Period

Initial considerations of how young children conceptualize race gained prominence in social commentary and academic literature through the historic legal case of Brown versus. the Board of Education (Bergner 2009). The work of Drs. Kenneth and Mamie Clark demonstrated that African American (Black) children perceived Euro‐American (White) children as possessing desirable traits such as intelligence, kindness, and beauty, at higher rates in comparison to Black children (Bergner 2009). These finding suggested that separating African American and Euro‐American children in educational settings caused emotional harm for the African American children and prevented them from truly having an equal education in comparison to their Euro‐American peers (Bergner 2009).

The Clarks' study sparked others to explore how children come to recognize and assign meaning to the construct of race through various processes of socialization (Hughes et al. 2006). The children in the Clarks' study ranged in age from 3 to 7 (Bergner 2009). Studies that followed often focused on older children and adolescents with a limited focus on children under the age of 3 years (e.g., Hughes et al. 2006; Priest et al. 2014; Raabe and Beelmann 2011; Skinner and Meltzoff 2019). Studies including infants and toddlers began to gain more prominence in the early 2000s with researchers investigating infants' visual preference when exposed to images of same‐race and other‐race faces. These studies demonstrated that young infants tended to prefer looking at images of same‐race faces (e.g., Bar‐Haim et al. 2006), while older infants began to show visual interest in other‐race faces (e.g., Katz 2003).

Researchers also explored infant's ability to visually discriminate between still images of faces with different phenotypic features. Study findings revealed that infants were able to demonstrate the capacity for this type of visual discrimination, but this capacity diminished over time with ethnic‐racial groups to which infants were not regularly exposed (e.g., Meissner and Brigham 2001). As the frequency of studies focused on infant preference and visual discrimination capacity increased, addition variables were included to test the responses of infants and toddlers to language differences and invitations given by same‐race and other‐race individuals to make choices or mimic behavior (e.g., Aktar et al. 2020; Njoroge et al. 2009; Xiao, Quinn, et al. 2018).

While studies on infant and toddler responses to ethnic‐racial differences have increased, the scope of research on ethnic‐racial awareness and factors influencing behavioral variation in this age group remains unclear. The Lifespan ERI study group that developed the lifespan model affirmed this gap in their paper entitled, *Persistent concerns: questions for research on ethnic‐racial identity development* (Rogers et al. 2020). In this paper, the authors addressed five persistent questions that emerged during the development of the lifespan model (Rogers et al. 2020). Both the first question—*When does ERI development begin and end*?—and the second—*How do we account for age‐dependent and conceptually initiated factors in ERI?* (Rogers et al. 2020, 131)?—highlight areas in this line of research that remain somewhat opaque. While some exemplar studies exist (e.g., Timeo et al. 2017), the extent to which ERI development has been studied in relation the ethnic‐racial priming period is unclear.

In addition to organizing ERI development by phases, the lifespan model also organizes it by dimensions. The first dimension of ERI development is “ethnic‐racial awareness [which] captures individuals' perceptions that ethnic‐racial groups are categories with social meaningfulness, as well as individuals' perceptions about how ethnic‐racial groups are viewed in society” (Rogers et al. 2020, 103). The dimension of ethnic‐racial awareness is developmentally relevant to infants and toddlers. Ethnic‐racial awareness starts during the ethnic‐racial priming period and presents differently across the lifespan with increased sophistication over time. Again, the extent to which the initial emergence of ethnic‐racial awareness has been studied among infants and toddlers is unknown. Given the relative ambiguity of ERI development research in the earliest developmental periods, there have been calls for additional research on ERI specifically within infancy and toddlerhood (e.g., Rogers et al. 2020; Ruck et al. 2021; Waxman 2021).

## 2 The Current Study

This study aims to contribute to the understanding of the early stages of the lifespan model by summarizing and mapping what is known about the development of ethnic‐racial awareness among children birth to 3 years of age and identifying potential gaps in the present literature. Specifically, this study examines (1) how ethnic‐racial awareness among infants and toddlers has been studied and (2) how caregivers’ ethnic‐racial socialization practices with infants and toddlers have been studied. Addressing these questions can deepen understanding of the ethnic‐racial priming period and inform future directions for research in this emerging area of study.

## 3 Method

The current study utilized a scoping review to systematically identify and analyze the existing literature on ethnic‐racial awareness, and influences on this awareness among infants and toddlers. Scoping reviews are a method to examine the state of a topical research area that is new or emerging (Mays et al. 2001). Unlike other advanced evidence syntheses such as systematic reviews and meta‐analyses, scoping reviews do not include in‐depth evaluation of research quality, quantitative analyses of study results, or extensive analysis of implications for policy or practice (Arksey and O’Malley 2005). Rather, they illuminate the breadth and depth of research in an area and identify existing gaps which may justify and guide more detailed systematic reviews or identify directions for further original research (Arksey and O’Malley 2005). Further, scoping reviews offer a systematic way to demarcate and map a body of knowledge in order to identify key concepts and determine appropriate next steps in moving a body of research forward (Mays et al. 2001; Tricco et al. 2018).

While previous reviews have included literature on ethnic‐racial awareness and potential influences, there is limited focus on children under the age of 3 years (e.g., Hughes et al. 2006; Priest et al. 2014; Raabe and Beelmann 2011; Skinner & Meltzoff 2019). Studies that do include infants and toddlers tend to emphasize child behavior that demonstrates the salience of ethnicity (e.g., looking time studies), yet the size and range of the research literature on the topic of ethnic‐racial awareness and the factors that might influence variation of behavior among infants and toddlers specifically is unclear. Thus, a scoping review was selected as the methodology for this study. In addition to examining the literature to determine the current scope of research on the development of ethnic‐racial awareness among infants and toddlers, this study also aimed to detect knowledge gaps in order to provide direction for future research on this topic.

The research team was comprised of five individuals, all of whom are co‐authors of this study. At the time the study was conducted, two of the team members (including the first author) were doctoral students, two were faculty members, one was a post doc researcher, and the final team member was a research librarian. The personal ERI’s of each team member at the time this research was conducted are documented in Table 1.

**TABLE 1:** Ethnic‐racial identities of research team members.

Research team member	Personal ethnic‐racial identity
Doctoral student	Black‐biracial with West African and Western European ancestry
Doctoral student	Korean
Post doctoral researcher	White Southern European
Associate professor	White European American
Professor	White with Northen European ancestry
Research librarian	White European American

The development of the research approach was guided by the PRISMA extension for scoping reviews (Tricco et al. 2018) and the framework proposed by Arksey and O'Malley (2005). Our review process followed the stages recommended by Arksey and O'Malley:

Stage 1 Identifying the research question.

Stage 2 Identifying relevant studies.

Stage 3 Study selection.

Stage 4 Charting the data.

Stage 5 Collating, summarizing, and reporting the results.

This scoping review is reported using the PRISMA‐ScR and PRISMA‐Searching guidance. An internal protocol was created but not registered.

### 3.1 Eligibility Criteria

The inclusion criteria used to determine which studies were included in this scoping review can be seen in Table 2.

**TABLE 2:** Inclusion criteria and descriptions.

Inclusion criteria	Descriptions
Published from January 1, 1990, through present.	The last database search for this study was completed on March 6, 2023.
Published in the English language.	Included studies had to be available in the English language due to English being the primary language used by all research team members.
Focus on the development of ethnic‐racial awareness among infants and toddlers.	Studies had to explicitly address how infants and toddlers develop conceptualizations of race and ethnicity, personal identity, visual categorization of people, in‐group and out‐group distinctions and preferences, and ethnic‐racial familiarity.
Focused on understanding race and ethnicity in relation to infants and toddlers 0–3 years (36 months but not beyond).	When studies include children within this age range and older children, they were only included when age could be explicitly disaggregated in the results.
Published and unpublished empirical studies and review articles such as meta‐analysis, and systematic reviews.	Studies in journal articles, books, and dissertations were included.
Descriptive and intervention studies using qualitative, quantitative, or mixed methods and had to include a direct measure of child behavior.	The research team was interested in including studies that provided parent‐report data and included the results of direct child observations. The reason was to specifically look at variation in child outcomes in combination with parent report data. The research team was interested in accounting for observable differences that could be seen in child behavior compared to potential influences on variation within child behavior. Parent report measures were only acceptable when parents were trained as observers of child behavior.

### 3.2 Information Sources

An initial search was run June 10th and 11th 2021 in seven databases: PsycInfo (ProQuest), PsycArticles (ProQuest, bundled with PsycInfo), Web of Science Core Collection, ERIC (ProQuest), Education Source (EBSCO), Sociological Abstracts (ProQuest), and Family and Society Studies (EBSCO). An updated search was run March 6th, 2023, in the same seven databases. The search strategies were designed, in consultation with the research team, by a research librarian who is a co‐author on this paper. Keywords selected captured the concepts of infants and toddlers, socialization, and race or ethnicity. The search string was as follows:

(Infan* OR toddler* OR baby OR babies) AND (Sociali* OR recogni* OR identity OR preference OR raciali?ation OR bias OR perception OR attitude OR categor*) AND (race OR ethni* OR racial). This search string was intended to capture any study that explicitly addressed how infants and toddlers develop conceptualizations of race, ethnicity, personal identity, visual categorization of people, in‐group/out‐group distinctions or preferences, and ethnic‐racial bias.

### 3.3 Search Strategy

Keywords were searched for in the titles and abstracts. Depending on the database, appropriate controlled vocabulary terms were selected from each database's thesaurus. Results were limited to articles from January 1, 1990, through March 6, 2023. No other limits were applied. Individual search strategies for each database can be found in Appendix A. This search yielded 7059 citations. 1613 duplicates were removed using the web‐based research platforms Covidence and Zotero.

**FIGURE 1:**
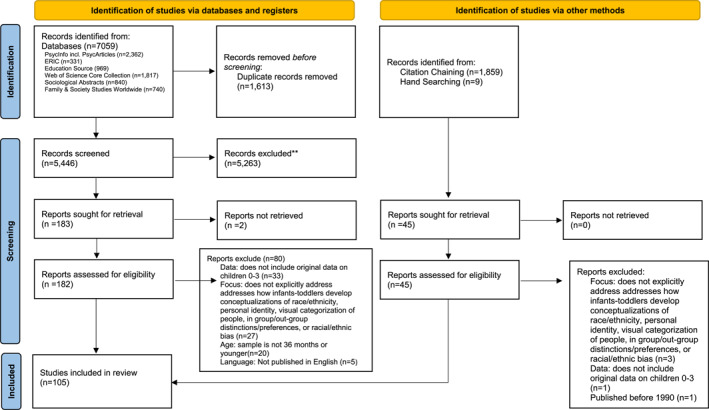
PRISMA 2020 flow diagram.

Citation chaining was performed on 8 seminal papers using the reference lists and Scopus or Google Scholar, which resulted in 2049 citations. 190 duplicates were removed. The manual process of looking through relevant journals to identify papers that met inclusion criteria known as hand searching (Richards 2008) added an additional 9 papers. After the removal of one duplicate, 1868 papers were added for the screening of citations via other methods. Two additional organizations, the National Black Child Development Institute, and the Abolitionist Teaching Network, were included in the pilot search, but were not found to have enough applicable information to be included in the final search. The complete PRISMA 2020 Flow Diagram is shown in Figure 1.

### 3.4 Selection of Evidence

A minimum of two research team members independently reviewed all titles and abstracts that were identified through the formal search process. Inclusion and exclusion criteria were used to select studies for full review. Any discrepancies in inclusion and exclusion criteria application were resolved by team discussion and consensus. Next, a minimum of two research team members independently reviewed the full text for all studies that were included after the title and abstract review. The same processes for applying inclusion and exclusion criteria and resolving discrepancies were utilized at this stage. After this screening process and the removal of duplicates between the search methods, 105 papers were included in the final analysis (see Appendix B).

### 3.5 Data Charting Process

The Covidence platform was utilized for screening and coding research studies published between January 1, 1990, and March 6, 2023. Covidence is designed to streamline the process of systematic reviews. This platform allows for independent coding by multiple reviewers, resolving coding conflicts by consensus, and populating coding results into a spreadsheet format. For the current study, Covidence was utilized in three phases. First, all reviewers independently screened the titles and abstracts that were identified through keyword database searchers and hand searching. Second, reviewers independently applied inclusion and exclusion criteria to full articles identified after the initial screening phase. Lastly, full articles that met all inclusion criteria were coded independently by reviewers. An internally developed coding form was utilized to code all included studies. During the coding process, the research team identified papers that presented more than one individual study, that is, those that reported multiple experiments with different samples within a single paper. When multiple studies were identified within a single paper, each study was coded separately. One hundred sixty‐eight unique studies were coded from within the 105 papers included in the sample.

### 3.6 Data Items

Variables were coded at either the paper or the study level using a “check all that apply” coding approach (see Table 3). Coding completed at the paper level included the entire journal article, book chapter, or dissertation, while coding at the unique study level included each separate experiment within an included journal article, book chapter, or dissertation. Type of publication, publication year, and discussion of potential influences on variation in the development of ethnic‐racial awareness among infants and toddlers were all variables coded at the paper level. The following variables were coded at the unique study level: study design, study setting, geographic location, sample demographics, study method, stimuli, outcome measures, and results. Additionally, the usage of any tools to measure influences on the development of ethnic‐racial awareness among infants and toddlers or ethnic‐racial socialization practices among their caregivers was also coded at the study level. Conflicts between independent coders were resolved by consensus during each phase.

**TABLE 3:** Coded categorical variables.

Variable	Categories coded	Number of studies
Variables coded at the paper level (*n* = 105) to answer RQ1		
Type of publication	Peer reviewed journal article	97
	Dissertation	5
	Open access journal article	2
	Book chapter	1
Year of publication	1990–2023	See Figure 2
Variables coded at the study level (*n* = 168) to answer RQ1		
Geographic location	Region of country or name of state or province if available	See Figure 2
Study design	Randomized experimental study	129
	Quasi‐experimental study	30
	Intervention study	4
	Naturalistic observational study	4
	Qualitative study	1
Study method	Single age or short age range study	105
	Cross sectional study	50
	Longitudinal study	13
Study setting	Lab	158
	Home	8
	Other	2
Number of participants	16–213	*M* = 53 (SD = 38)
Race and ethnicity of participants	Black	See Figure 3
	Asian	
	Native	
	Latiné or Latinx	
	White	
	Multiracial	
Age of participants (in months)	Newborn to 36‐month	See Figure 4
Stimuli	Still face images	96
	Language	51
	Videos	32
	Actors	14
	Dynamic images	19
	Toys, objects, or food	5
	Food	5
Variables coded at the study level (*n* = 168) to answer RQ1		
Outcome measures	Looking time: Length of time spent looking at stimuli or eye gaze: Specific area where gaze is focused	134
	Object choice: Child choosing a toy or object	14
	Food choice: Child choosing a food	
	Brain activity: Any measurement of brain activity	8
	Imitation: Child consciously imitating another (e.g., lifting arms up)	4
	Mimicry: Child subconsciously mimicking another (e.g., pupil size changing)	3
	Both looking time and brain activity	5
Results	Demonstrated capacity to distinguish others based on phenotypic features or language	133
	Demonstrated in‐group familiarity when distinguishing others	100
	Demonstrated out‐group interest	17
Results	Distinguished differences without demonstrating familiarity or preference for either an out‐group or an in‐group	18
Variables coded at the study level for studies that did not include in‐group stimuli (*n* = 24) to answer RQ1		
Results	Distinguished between groups using other characteristics such as facial attractiveness, language, and shared features (i.e., clothing colour)	20
	Did not distinguish between groups using other characteristics	2
Variables coded at the paper level (*n* = 105) to answer RQ3		
Discussion of ethnic‐racial socialization influences	Not applicable because ethnic‐racial socialization factors were not discussed	52
	Exposure to other‐race individuals	39
	Exposure to other languages	9
	Exposure to diverse materials (e.g., books, toys)	1
	Exposure to diverse media (e.g., television shows, apps)	0
	Exposure to diverse cultural experiences (e.g., food, music, celebrations)	0
	Other (living in neighborhoods that are ethnically and racially diverse or linguistically diverse based on census data)	4
Ethnic‐racial socialization measures (name and description of measurement tool used)	Non‐validated, parent questionaries	14
	Census data	4
	Language exposure questionnaire Bosch & Sebastian‐Galles, 1997	5
	Scale adapted from Brown et al. (1999) assessing participants familiarity with African people	1
	Infant‐individual interaction Scale	1
	Infant‐caregiver and family member interaction Scale	1

**FIGURE 2:**
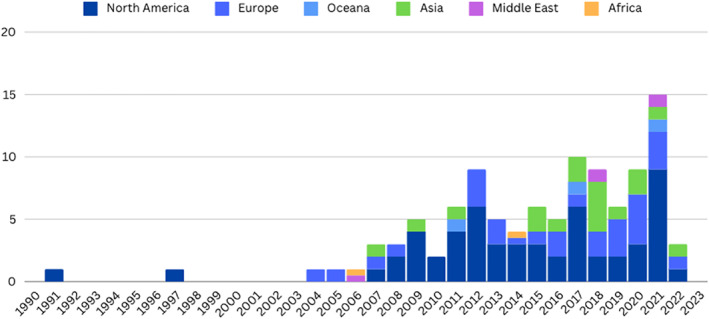
Publication of included studies by year and geography.

**FIGURE 3:**
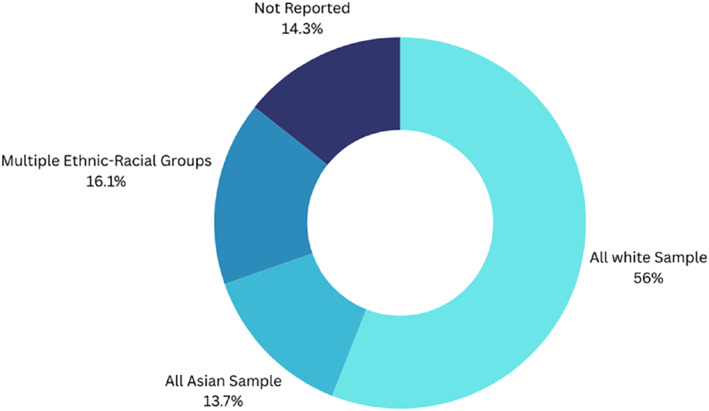
Ethnic‐racial diversity among study samples.

**FIGURE 4:**
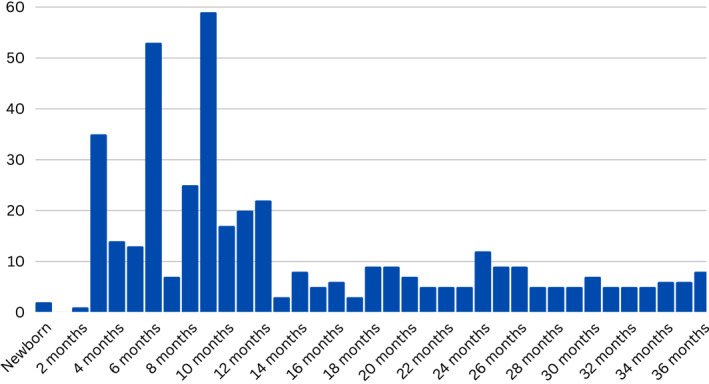
Number of studies including each age group.

## 4 Findings

The final analysis included 105 papers published between January 1990 and March 2023. Included papers reported on a total of 168 unique studies. Included studies explicitly address how infants and toddlers (birth to age 3) use visual and auditory stimuli to categorize others, develop conceptualizations of in‐group and out‐group distinctions, and establish ethnic‐racial familiarity.

### 4.1 How has Ethnic‐Racial Awareness Among Infants and Toddlers Been Studied?

#### 4.1.1 Publication Type, Publication Year, and Study Location

Out of the 105 included papers, 93% (*n* = 97) were articles published in peer reviewed journals. Five dissertations, 2 open access journal articles, and 1 book chapter were also included. Findings for the paper publication year demonstrated how interest in this research topic has grown in recent years. Only 11 papers meeting the inclusion criteria for this scoping review were published during the first 19 years (between January 1990 and December 2008) of this scoping review's 34‐year range. The number of relevant studies began to increase in 2009 with 94 papers meeting the inclusion criteria published between January 2009 and March 2023.

The vast majority of studies were conducted in the United States (*n* = 76) followed by Canada (*n* = 21), Germany (*n* = 14), the UK (*n* = 13), China (*n* = 10), France (*n* = 8), Japan (*n* = 6), Singapore (*n* = 5), and Israel (*n* = 5). A smaller number of studies were conducted in the following countries: Australia (*n* = 3), Spain (*n* = 2), Netherlands (*n* = 2), Ethiopia (*n* = 1), Cameroon (*n* = 1), and Taiwan (*n* = 1). Two studies included participants from more than one country within their sample populations; one study was conducted in both Germany and Cameroon, and another study was conducted in both Israel and Ethiopia. As seen in Figure 2, these findings reveal the disproportionate focus of this research in Western countries.

#### 4.1.2 Study Design and Research Method

The majority of the 168 included studies utilized randomized experimental designs (*n* = 129). Fewer studies used quasi‐experimental designs (*n* = 30), interventions (*n* = 4), naturalistic observation (*n* = 4), and qualitative designs (*n* = 1). Nearly all studies were conducted in lab settings (*n* = 158), with 8 additional studies taking place in the home setting and 2 studies being conducted in a school setting.

While 50 studies used a cross‐sectional approach, most studies included a single age or a single short age range for their sample (*n* = 105), and only 8% of studies utilized a longitudinal design (*n* = 13). Among the few longitudinal studies, 62% had samples that included only White participants, and only 3 studies included toddlers. All three of these studies were published by Katz and colleagues in the 1997 book: *Race, gender and young children*.

#### 4.1.3 Sample Demographics

The average sample size among included studies was 53 (SD = 38) participants with the smallest sample size being 16 participants and the largest sample size being 213 participants.

Most studies included sample populations from single ethnic‐racial groups (*n* = 117), and the majority involved White participants exclusively (*n* = 94), followed by Asian participants exclusively (*n* = 23). A smaller number of studies included multiple ethnic‐racial groups (*n* = 27), and only 10 studies included biracial or Multiracial participants in their samples. Of the studies that did not report the ethnic‐racial demographics of their sample populations (*n* = 24), all were conducted in countries with majority white populations: the USA (*n* = 18), the UK (*n* = 3), Spain (*n* = 2), and Germany (*n* = 1). These study locations suggest that the sample populations were likely all or majority White (Figure 3).

Further, infants were more likely to be included in the study samples compared to toddlers. Children 12 months of age and younger were included in the majority of the studies (*n* = 134) compared to the number of studies that included toddlers ages 13–36 months (*n* = 30). Only four studies included both children 12 months or younger and children 13–36 months of age. The most common age of study participants was 9 months (*n* = 59) followed by 6 months (*n* = 53), and 3 months (*n* = 35). A full summary of the ages included in the sample populations can be seen in Figure 4.

#### 4.1.4 Stimuli and Outcome Measures

The majority (*n* = 124) of the 168 individual studies examined how infants distinguish others based on observable, phenotypic differences such as skin tone, facial features, and emotional expressions. An additional 27 studies examined how infants and toddlers distinguish others based on language and accent, and 17 studies included both observable, phenotypic differences and language or accent differences as examined variables. While some may think of language as separate from the constructs of ethnic‐racial awareness and ethnic‐racial identity, it is widely considered to be a significant element of ethnic‐identity among many ethnic‐racial groups (e.g., DeJesus et al. 2019).

Still face images were used as stimuli in most of the studies (*n* = 96) followed by vocal or sound stimuli (*n* = 51). Other stimuli included videos (*n* = 32), dynamic facial images (*n* = 19), live actors (*n* = 14), and objects such as toys or food (*n* = 5). The vast majority of studies utilized looking time as an outcome measure (*n* = 134). Some studies measured brain activity (*n* = 8), and 5 studies used both looking time and brain activity as the outcome measures. Other studies used object choice (*n* = 14), and fewer studies used behavior imitation (*n* = 4) or pupil dilation mimicry (*n* = 3) as outcome measures. Less frequently, studies have examined children's object choices and behavior (10%), brain activity (4%), behavior imitation (2%), and pupil dilation mimicry (2%) as responses indicating development of ethnic‐racial awareness.

#### 4.1.5 Study Results

The variable categories for results included ability to distinguish faces based on distinct features, same‐race familiarity or preference, other‐race familiarity or preference, ability to distinguish between own‐language and other‐language, same‐language familiarity or preference, or anticipation of behavior based on in‐group or out‐group membership. Most included studies reported average findings for the whole sample with little attention paid to individual differences or potential influences on or predictors of variation in the development of early ethnic‐racial awareness. However, potential predictors of variation can be detected when looking across the sample characteristics of included studies, most predominantly, age.

Of the 168 included studies, only 9 studies did not demonstrate infant and toddler capacity to distinguish others based on phenotypic features or language. Two of these studies were conducted with newborns suggesting that the capacity to distinguish people based on different facial features is not present at birth (D. Kelly et al. 2005; Quinn et al. 2008). Two additional studies from Kinzler and colleagues (Kinzler and Spelke 2011) showed that White 10‐month‐olds equally gave a toy to a Black or a White actor, and White 2.5–3‐year‐olds equally gave a gift to a Black or a White actor. Similarly, Castelli and colleagues found that White 1–2‐year‐olds took food equally from a Black or White person, but White 3–4‐year‐olds were more likely to take food from a White person (Castelli and Carraro 2020). Taken together, these findings suggest that age is one influence on variation in the development of ERI awareness.

A study by Hayden et al. (2012) showed that White 3.5‐month‐olds were not able to discriminate between own‐race and other‐race faces when the facial images were inverted. This result seemed to be related to the way the faces were displayed as participants were able to discriminate between the same own‐race and other‐race facial images when they were not inverted (Hayden et al. 2012). Thus, the quality and context of exposure may be another influence on variation in the development of ERI awareness.

The final three studies in this category demonstrated conflicting findings compared to other studies with similar samples and designs. In a 2014 study by Howard and colleagues, 19‐month‐old native English speakers did not show imitation preferences between English or Spanish speaking actors (Howard et al. 2014). A 2017 study found that 4–12‐month‐old infants did not show preferential looking time toward images of same and other race faces (Montoya et al. 2017). Finally, a study by Xiao and colleagues showed that 7‐month‐olds followed the gaze of same and other‐race adults equally when they gave consistently reliable visual cues (Xiao, Wu, et al. 2018). The results of these three studies demonstrate the need to continue exploring the differences in context and variables that impact the development of ethnic‐racial awareness among infants and toddlers.

#### 4.1.6 Results for Studies That Included in‐Group Stimuli

Of the 144 studies that included in‐group stimuli (stimuli with features such as skin color or language that are the same as participants), most (*n* = 133) demonstrated that infants and toddlers are able to distinguish others based on differences they observe (i.e., phenotypic features) or hear (i.e., language or accent). Further, the majority of studies that included in‐group stimuli demonstrated that infants and toddlers display in‐group familiarity (e.g., higher proportion of looking time when encountering in‐group stimuli) when distinguishing others (*n* = 100).

While most studies demonstrated that infants and toddlers are more drawn to faces and languages that are most familiar to them, fewer studies demonstrated that infants and toddlers display out‐group interest (e.g., higher proportion of looking time when encountering out‐group stimuli) when presented with both in‐group and out‐group stimuli (*n* = 17). Additionally, 18 studies showed that infants and toddlers can distinguish differences even though they do not demonstrate familiarity or preference for either an out‐group or an in‐group (e.g., looking time indicates ability to distinguish difference, but proportion of looking time is relatively equal toward both in‐group and out‐group stimuli). This group of studies highlights the need for additional research that explores influences that may be associated with variation in how young children respond to people who represent out‐groups.

#### 4.1.7 Results for Studies That Did not Included in‐Group Stimuli

Some studies did not include in‐group stimuli (*n* = 24). Among these studies, most (*n* = 22) had results that demonstrated infants and toddlers' ability to distinguish between groups using other characteristics such as facial attractiveness, language, and shared features (i.e., clothing color). Two of the studies that did not include in‐group stimuli reported that participants were not able to distinguish between groups using other characteristics. One of these studies used facial images of two other‐race groups (Black and Asian) as stimuli. After being habituated to select Black and Asian faces, White 9‐month‐olds who were habituated to select Black faces were not able to distinguish a novel Asian face (Quinn et al. 2015). Likewise, the infants did not demonstrate the ability to distinguish a novel Black face if they were habituated to Asian faces (Quinn et al. 2015). These findings suggest that the participating infants may have perceived both Black and Asian faces as a single other‐race group (Quinn et al. 2015). However, the results of this particular experiment were null, which makes interpretation unclear (Quinn et al. 2015).

The other study that demonstrated an inability of participants to distinguish between groups used animated geometric objects as the stimuli (Powell and Spelke 2013). The objects represented belonging to distinct groups based on having similar features. The objects were also presented with different features, colors and shapes (Powell and Spelke 2013). While an outlier since the study did not use human faces as the stimuli, this study met inclusion criteria as it involved direct measures of infant categorizing behavior based on visual perception and measured by looking time (Powell and Spelke 2013). Other studies within the same paper, found that when identical objects (e.g., same color and shape) were paired with other social cues such as proximity on the screen and synchronous movement, 8‐month‐olds could distinguish the groups, and they demonstrated anticipation of similar behavior based on group membership. However, the infants did not demonstrate this ability to distinguish groups in another study where the social cues such as proximity and synchronous movement were absent (Powell and Spelke 2013). While providing interesting information related to how infants determine group membership and predict group behavior, this particular set of studies is inconsistent with the others in its use of animated geometric objects as stimuli.

#### 4.1.8 Ethnic‐Racial Socialization Measures

Influences on the development of ethnic‐racial awareness among infants and toddlers have primarily been studied using non‐validated questionnaires that ask caregivers to indicate infant and toddler exposure to other ethnic‐racial groups (*n* = 39) and other languages (*n* = 9). Ethnic‐racial diversity (*n* = 3) and linguistic diversity (*n* = 1) of geographic locations based on census data have also been used to measure possible influences.

### 4.2 How Have Caregivers’ Ethnic‐Racial Socialization Practices With Infants and Toddlers Been Studied?

In this scoping review, ERS messages were considered a potential influence of variation in ERI awareness development among infants and toddlers. We specifically coded for whether or not ERS influences were mentioned in the discussion, implications, or conclusion sections of included papers. The search string used for this scoping review included: (Infan* OR toddler* OR baby OR babies) AND (Sociali* OR recogni* OR identity OR preference OR raciali?ation OR bias OR perception OR attitude OR categor*) AND (race OR ethni* OR racial). The breadth of this search string ensured that all papers addressing ERS of infants and toddlers were identified. ERS influences mentioned were only coded if they were presented as factors that could explain variation in the study outcomes. The coding protocol also included recording any specific methods or assessment tools used to measure ERS socialization or ERS influences. If socialization influences were measured, the name or description of the measurement tool(s) was documented on the coding protocol.

Findings from this scoping review demonstrated that engagement in ERS practices among caregivers of infants and toddlers has generally not been studied. In fact, no studies in this scoping review included organic engagement in ERS practices as a variable of interest, and only one study (Heron‐Delaney et al. 2011) involved caregiver engagement in intentional exposure to other‐race images as part of the study design. While no papers in our sample provided direct examples of caregiver engagement in ERS, there were two promising findings that could inform future intervention work. A longitudinal study conducted by Heron‐Delaney and colleagues in 2011 found that the ability to discriminate between other‐race faces was retained among White infants after they were regularly exposed to a set of books showing diverse faces between 6‐and‐9‐month of age (Heron‐Delaney et al. 2011). Similarly, Anzures et al. (2012) found that 8—10‐month‐old White infants who had daily experience with Asian faces over three weeks were less likely to demonstrate perceptual narrowing with respect to Asian faces. Both studies provide possible directions for investigating how the development of ethnic‐racial bias might be disrupted during infancy and toddlerhood.

## 5 Discussion

### 5.1 Present State of Research

The results of this scoping review highlight a growing body of research focused on ethnic‐racial awareness in early childhood. Existing studies contribute to our understanding of the ethnic‐racial priming period within the lifespan model, while also underscoring the continued need for research specifically addressing the earliest developmental phase. This review found substantial evidence supporting the developmental relevance of ethnic‐racial awareness among infants and toddlers. Notably, 92% of studies reported that infants and toddlers can differentiate individuals based on phenotypic differences in visual and auditory features and show signs of in‐group familiarity. Among studies that included in‐group stimuli, 69% indicated that infants and toddlers demonstrate familiarity with in‐group characteristics such as ethnicity, race, language, or culture. However, methodological limitations and the frequent demographic homogeneity of study samples point to critical gaps in the current literature and signal the need for more diverse and rigorous approaches to studying this early developmental period.

Relative to study design, randomized experimental designs in lab settings with looking time as the most dominant behavioral measure have been the most used methodologies. While lab‐based studies allow for the establishment of a more controlled study environment, the lack of studies that take place in more naturalistic settings, and the near complete omission of qualitative methods limits our understanding of how ethnic‐racial awareness emerges and is expressed among young children within the context of daily life. Use of still face images and looking‐time behavior provides consistent standardization; however, it is important to note that infants and toddlers will primarily interact with others in dynamic ways that may not be best reflected by the static images of photographs.

This scoping review also illuminated a remarkable gap in terms of examining ethnic‐racial awareness and developmental influences among infants from minoritized populations and among toddlers (13–36 months). The sample populations in the included studies were overwhelmingly White children who were 12 months of age or younger. The imbalance of ethnic‐racial diversity and the limited inclusion of toddlers demonstrates that the findings within this body of research to date primarily measure outcomes for White infants. Further, the majority of studies were conducted in what Henrich et al. (2010) refer to as WEIRD societies—Western, Educated, Industrialized, Rich, and Democratic. Thus, there is a gap in what is known about the development of ethnic‐racial awareness among infants and toddlers in the global south.

Findings also demonstrated an absence of studies examining early anti‐bias interventions and ethnic‐racial socialization practices among caregivers of infants and toddlers. While not all studies demonstrated infant and toddler capacity to distinguish others based on race, prior research does suggest that a bias toward Whiteness may not be prominent in infants and toddlers but has been observed in the preschool and early elementary years (Castelli and Carraro 2020; Kinzler and Spelke 2011). This implies that efforts to disrupt the development of harmful bias among young children may have a window of opportunity in the infant and toddler years. Meaningful evaluation of interventions will require longitudinal study designs, however; few studies employed longitudinal designs (*n* = 13) and less than 2% of the included longitudinal studies included toddlers in their sample.

### 5.2 Implications for the Lifespan Model of Ethnic‐Racial Identity

The lifespan model clearly situates the origins of ERI within early infancy and touts its value as being a framework that organizes an otherwise disjointed collection into a connected model that threads all ERI developmental periods together. While an immensely helpful framework, understanding how ERI development happens across the lifespan within the dimensions that the lifespan model presents is essential in connecting theory to practice. This scoping review sought to address some specific questions that would also further illuminate what is known about the ethnic‐racial priming period. The findings from this scoping review clarified the capacities that infants and toddlers bring to the ERI development process and helped to operationalize the lifespan model's first dimension of ethnic‐racial awareness as it occurs within the ethnic‐racial priming period of birth to age 3. Perceptions are emerging and preferences may connect to initial understandings of social meaningfulness of ERI categories.

Relative to the persistent questions presented by Rogers and colleges (2020), this scoping review addresses the first part of the first question—When does ERI development begin…?—by demonstrating that it begins in the very first months of life. The scoping review's implications for the lifespan model as also evident in how it can inform future research that will continue to compliment and strengthen the model in its ability to be operationalized and applied. “This scoping review also addressed the question—*How do we account for age‐dependent and conceptually initiated factors in ERI?* (Rogers et al. 2020, 131). Findings indicate a need for further research to answer this question, particularly due to the lack of longitudinal studies both within and beyond the ethnic‐racial priming period, and the essential non‐existence of studies focused on the ERS practices among caregivers of infants and toddlers.

### 5.3 Additional Implications for Research

This scoping review confirmed that ethnic‐racial awareness emerges in infancy. While this was already known, results from this scoping review explicitly demonstrated that this knowledge is primarily based on findings from looking time studies done with very young, White, infants. Looking time studies do not replicate the dynamic interactions infants have with people in their environments. Thus, there is a need for future research to focus on the inclusion of observational studies that are conducted in settings that more closely mimic the natural environment of infants and mirror the ways in which they engage with individuals in those environments. Naturalistic observation methods are vital when studying young children before they acquire verbal language and pointing skills (Marcella and Howes 2015). Additional emphasis on research in naturalistic settings could also support more understanding of individual differences in ethnic‐racial awareness, familiarity, and preferences among children birth to 3 years old, which might be best explored in naturalistic settings.

There is also a need to increase the ethnic‐racial diversity of participants when studying ethnic‐racial awareness and developmental influences among infants and toddlers. The U.S. population is simultaneously increasing in ethnic‐racial diversity and demonstrating more ethnic‐racial bias. Experts predict that Gen Z will be the last generation that is majority White in the United States (Frey 2023). Further, the Multiracial population is a rapidly expanding demographic group (Nishina & Witcow 2020). This is especially evident among child population in the United States (Lopez et al. 2015). Continuing to conduct this research primarily with populations that are exclusively White or only including single ethnic‐racial groups in study samples severely limits the expansion of knowledge in the field. This scoping review demonstrated that even when study participants were not from only one ethnic‐racial demographic group, samples that included two ethnic‐racial demographic groups were typically comprised of participants who were white and participants who were Black. Given the shifting demographics globally and specifically in the U.S. context, this dyadic approach is outdated and incomplete. Additional ethnic‐racial groups should be included in future research with greater frequency. This need is especially notable with Multiracial populations.

These findings are aligned with the core issues identified by Singh et al. (2023) in their review of infant research conducted between 2011 and 2022. Their review found a lack of ethnic‐racial diversity among study participants in infant research and a lack of inclusivity in terms of location of studies and methodological approaches (Singh et al. 2023). Similarly to this scoping review, Singh et al. (2023), found that the majority of infant research studies included in their review had all White samples (or no mention of ethnic‐racial demographics), and utilized methodologies designed for lab settings.

Lack of diversity among study participants was also illuminated in this scoping review with the toddler age group being under‐represented in the ethnic‐racial awareness development literature. The fact that toddlers are not frequently included in samples of studies on this topic excludes a key period along the developmental continuum. This is especially problematic given that the second year of life is characterized by the emergence of a sense of self as distinct from others (Rochat 2003). Future research would benefit from a more purposive inclusion of toddler participants between 12 and 36 months of age.

Finally, there is a clear need for future research to better measure the ethnic‐racial socialization practices that caregivers of infants and toddlers engage in and their impact on child development. While data on potential developmental influences on ethnic‐racial awareness (such as exposure to ethnically and racially diverse individuals) are being collected through parent report, there is a lack of valid measures to examine ERS practices and outcomes with the infant and toddler age group. Future research should investigate what types of ERS infants and toddlers are being exposed to at what levels and how they are responding. Along with the use of validated measures, more longitudinal intervention studies will be needed to understand the impact of ERS on infants and toddlers and to test the results of interventions designed to promote healthy ethnic racial identity and disrupt the development of ethnic‐racial bias in the earliest years.

### 5.4 Limitations

While providing an important summary of the extant literature on early ethnic‐racial awareness and developmental influences among infants and toddlers, this scoping review also has some limitations. First, the search was done with databases that focused on psychological research, and did not include research that may have been done in other social sciences relevant to this topic, such as anthropology. The specific time frame is also a limitation. Results from this scoping review include papers published from January 1, 1990, through March 6, 2023. Any studies examining the developmental relevance of ethnicity and race among infants and toddlers that have been published after that point are not included in this scoping review.

A scoping review is limited by design and does not include statistical analysis. This methodology is not intended to synthesize results nor appraise the quality of included evidence (Arksey and O’Malley 2005), but rather, to offer a first step toward a synthesized understanding of a burgeoning research area. Within these limitations, this scoping review described the scope of foundational knowledge on early ethnic‐racial awareness and identified several specific gaps that can be used to inform future research.

## 6 Conclusions

The goal of this scoping review was expand understanding of the ethnic‐racial priming period within the lifespan model by systematically identifying and analyzing the existing literature on the development of ethnic‐racial among infants and toddlers. Ethnicity and race are clearly salient to infants and toddlers; however, the approach to studying early awareness of ethnicity and race has been quite narrow in terms of both sample populations and study methodologies. As a result, many questions about the ethnic‐racial priming period remain unanswered. Additionally, the lack of ethnic‐racial diversity in sample populations, the limited representation of global regions, and the dominance of Western‐centered research practices deeply limit the scope of our knowledge in this topical area and effectively constrain the ethical generalizability of findings (Singh et al. 2023).

Further, current research suggests that the first 3 years of life may provide a unique developmental window during which intentional ERS practices may be highly influential. There may be opportunities to disrupt the development of harmful ethnic‐racial biases that can evolve from a lack of intentional guidance as infants use early perception skills to sort and categorize familiar and unfamiliar ethnic‐racial groups. This is especially relevant in the ethnic‐racial priming period as environment context and exposure can influence developmental trajectories during that period (Williams et al. 2020). Yet, how caregivers might intentionally practice ERS in the earliest years and the results of those practices on individual differences in development might produce are essentially unexplored areas of research.

Building on the call to engage in practices that “redress the current imbalance in participant representation,” from Singh et al. (2023, 715), intentional effort is needed to diversify both who is included in research studies on early ethnic‐racial awareness and how those studies are conducted. This scoping review also demonstrates a need for more research to expand our understanding of the lifespan model's ethnic‐racial priming period and clarify how much infants and toddlers can distinguish between and associate meaning with different ethnic‐racial identities. Moreover, additional studies are needed to identify the specific developmental trajectory of ethnic‐racial awareness in association with age and individual differences that might be predicted by measurable influences. These gaps should serve as guidance for future systematic reviews and research studies on the development of ethnic‐racial awareness among infants and toddlers during the ethnic‐racial priming period of the lifespan model.

## Author Contributions

**Anissa L. Eddie:** conceptualization, data curation, formal analysis, methodology, project administration, visualization, writing – original draft, writing – review and editing. **Claire D. Vallotton:** conceptualization, data curation, methodology, supervision, visualization, writing – review and editing. **Holly Brophy‐Herb:** data curation, writing – review and editing. **Loria Kim:** data curation, writing – review and editing. **Carin Graves:** conceptualization, methodology, software, supervision, writing – review and editing. **Danielle Dalimonte‐Merckling:** data curation.

## Ethics Statement

The authors have nothing to report.

## Consent

The authors have nothing to report.

## Conflicts of Interest

The authors declare no conflicts of interest.

## Data Availability

Data to support this research is available upon request.

## Appendix A Scoping Review Search Strategy.

Table A1.

**TABLE A1:** Scoping review search strategy.

# of search	Keywords and controlled vocabulary	# of results
*PsycInfo Including PsycArticles (ProQuest)* *Searched 3/6/23* *Keywords anywhere except full text*		
1	Infan* OR toddler* OR baby OR babies	186,144
2	MAINSUBJECT.EXACT.EXPLODE(“Early Childhood Development”)	40,806
3	1 OR 2	197,109
4	Sociali* OR recogni* OR identity OR preference OR raciali?ation OR bias OR perception OR attitude OR categor*	1,771,792
5	MAINSUBJECT.EXACT(“Socialization”) OR MAINSUBJECT.EXACT(“Racial Bias”) OR MAINSUBJECT.EXACT(“Preferences”) OR MAINSUBJECT.EXACT(“Self Concept”) OR MAINSUBJECT.EXACT(“Face Perception”) OR MAINSUBJECT.EXACT(“Racial and Ethnic Attitudes”) OR MAINSUBJECT.EXACT(“Social Categorization”)	141,812
6	4 OR 5	1,811,591
7	Race OR ethni* OR racial	215,633
8	MAINSUBJECT.EXACT(“Racial Identity”) OR MAINSUBJECT.EXACT.EXPLODE(“Ethnic Identity”)	20,522
9	7 OR 8	215,633
10	3 AND 6 AND 9	2582
11	3 AND 6 AND 9 1990‐present	2362
ERIC (ProQuest) Searched 3/6/23 Keywords anywhere except full text		
1	Infan* OR toddler* OR baby OR babies	28,405
2	MAINSUBJECT.EXACT.EXPLODE(“infants”) OR; MAINSUBJECT.EXACT(“Toddlers”)	18,626
3	1 OR 2	28,596
4	Sociali* OR recogni* OR identity OR preference OR raciali?ation OR bias OR perception OR attitude OR categor*	539,824
5	MAINSUBJECT.EXACT(“Visual Perception”) OR MAINSUBJECT.EXACT(“Preferences”) OR MAINSUBJECT.EXACT(“Racial Bias”) OR MAINSUBJECT.EXACT(“Racial Identification”) OR MAINSUBJECT.EXACT(“Socialization”) OR MAINSUBJECT.EXACT(“Race Attitudes”)	34,457
6	4 OR 5	546,017
7	Race OR ethni* OR racial	99,984
8	MAINSUBJECT.EXACT(“Race”) OR MAINSUBJECT.EXACT.EXPLODE(“Ethnic Groups”)	47,443
9	7 OR 8	120,056
10	3 AND 6 AND 9	459
11	3 AND 6 AND 9 1990‐present	331
Education Source (EBSCO) Searched 3/6/23		
1	Infan* OR toddler* OR baby OR babies	70,999
2	DE “Child psychology OR DE “Infant psychology” OR DE “Child Development”	52,164
3	1 OR 2	115,832
4	Sociali* OR recogni* OR identity OR preference OR raciali?ation OR bias OR perception OR attitude OR categor*	643,604
5	DE “Visual perception” OR DE “Perception” OR DE “Affiliation (Psychology)” OR DE “Attitude (Psychology)” OR DE “Group identity” OR “Attitudes of Ethnic Groups”	46,092
6	4 OR 5	643,821
7	Race OR ethni* OR racial	155,553
8	3 AND 6 AND 7	1136
9	3 AND 6 AND 7 1990‐present	969
Web of Science Core Collection (Web of Science) Searched 3//6/23		
1	TI (Infan* OR toddler* OR baby OR babies)	280,701
2	AB = (Infan* OR toddler* OR baby OR babies)	382,409
3	1 OR 2	544,947
4	TI = (Sociali* OR recogni* OR identity OR preference OR raciali?ation OR bias OR perception OR attitude OR categor*)	1,082,048
5	AB = (Sociali* OR recogni* OR identity OR preference OR raciali?ation OR bias OR perception OR attitude OR categor*)	4,191,834
6	4 OR 5	4,656,216
7	TI = (Race OR ethni* OR racial)	205,285
8	AB = (Race OR ethni* OR racial)	407,967
9	7 OR 8	532,193
10	3 AND 6 AND 9	1819
11	3 AND 6 AND 9 1990‐present	1817
Sociological Abstracts (ProQuest) Searched 3/6/2023 Keywords anywhere except full text		
1	Infan* OR toddler* OR baby OR babies	18,038
2	MAINSUBJECT.EXACT.EXPLODE(“Infants”) OR MAINSUBJECT.EXACT(“Preschool children”)	5321
3	1 OR 2	19,780
4	Sociali* OR recogni* OR identity OR preference OR raciali?ation OR bias OR perception OR attitude OR categor*	551,902
5	MAINSUBJECT.EXACT(“Socialization”) OR MAINSUBJECT.EXACT(“Attitudes”) OR MAINSUBJECT.EXACT(“Perceptions”) OR MAINSUBJECT.EXACT(“Self Concept”) OR MAINSUBJECT.EXACT(“Prejudice”) OR MAINSUBJECT.EXACT(“Internalization”) OR MAINSUBJECT.EXACT(“Recognition (Psychology)”) OR MAINSUBJECT.EXACT(“Bias”)	81,667
6	4 OR 5	563,350
7	Race OR ethni* OR racial	252,077
8	MAINSUBJECT.EXACT(“Ethnic Identity”) OR MAINSUBJECT.EXACT(“Ethnicity”) OR MAINSUBJECT.EXACT(“Race”)	66,560
9	7 OR 8	252,077
10	3 AND 6 AND 9	923
11	3 AND 6 AND 9 1990‐Present	840
Family and Society Studies Worldwide (EBSCO) Searched 3/6/23		
1	Infan* OR toddler* OR baby OR babies	112,978
2	Sociali* OR recogni* OR identity OR preference OR raciali?ation OR bias OR perception OR attitude OR categor*	253,858
3	Race OR ethni* OR racial	58,118
4	1 AND 2 AND 3	877
5	1 AND 2 AND 3 1990‐Present	740

## Appendix B List of Papers Included in the Scoping Review With Key Characteristics.

Table A2.

**TABLE A2:** Papers included in the scoping review with key characteristics.

Publication year	Country	Age of sample	Reference
1991	USA	6 months	Langlois et al. (1991)
1997	nr	6–36 months	Katz and Kofkin (1997)
2004	France	3 months	Sangrigoli and de Schonen (2004)
2005	UK	Newborns	D. Kelly et al. (2005)
2006	Israel	3 months	Bar‐Haim et al. (2006)
2007	nr	3.5 months	Hayden et al. (2007)
2007	UK	3, 6, 9 months	D. J. Kelly et al. (2007)
2007	China	3 months	D. J. Kelly et al. (2007)
2008	Germany	3, 12, 18 months	Schug (2009)
2008	nr	2, 5, 8, 11 months	Rennels and Davis (2008)
2008	nr	3 months	Quinn et al. (2008)
2009	USA	12 months	Shutts et al. (2009)
2009	nr	8 months	Ferguson et al. (2009)
2009	USA	6–36 months	Njoroge et al. (2009)
2009	China	3, 6 9 months	D. J. Kelly et al. (2009)
2009	nr	9 months	Hayden et al. (2009)
2010	nr	6, 9 months	Anzures et al. (2010)
2010	nr	6 months	Scott and Monesson (2010)
2011	Australia	6, 9 months	Heron‐Delaney et al. (2011)
2011	nr	9 months	Balas et al. (2011)
2011	nr	6–10 months	Wheeler et al. (2011)
2011	nr	10 months, 2.5–3 years	Kinzler and Spelke (2011)
2011	nr	6, 9 months	Anzures et al. (2011)
2011	nr	4–9 months	S. Liu et al. (2011)
2012	nr	11.5 months	Mahajan and Wynn (2012)
2012	nr	6, 9 months	W. S. Xiao et al. (2013)
2012	nr	6 months	Uttley et al. (2013)
2012	nr	3 months	Ziv (2013)
2012	nr	5, 9 months	Vogel et al. (2012)
2012	USA	3 months	Gaither et al. (2012)
2012	nr	8–10 months	Anzures et al. (2012)
2012	Germany	3 months, 6 months	Fassbender et al. (2012)
2012	nr	3.5 months	Hayden et al. (2012)
2013	nr	3–4 months, 9–10 months	Rennels and Cummings (2013)
2013	USA	8 months, 12 months	Powell and Spelke (2013)
2013	Germany	14 months	Buttelmann et al. (2013)
2013	USA	9 months	Kim (2014)
2013	Germany	3, 6, 9 months	Spangler et al. (2013)
2014	USA	19 months	Howard et al. (2014)
2014	nr	6 months, 9 months	W. S. Xiao et al. (2014)
2014	Cameroon	3 months, 6 months	Fassbender et al. (2014)
2014	nr	15 months	Burns and Sommerville (2014)
2015	USA	19 months	Howard et al. (2015)
2015	nr	3–4, 9–10, 12 months	Kayl (2015)
2015	USA	3, 10 months	Kim et al. (2015)
2015	UK	3–4, 8–9 months	Tham et al. (2015)
2015	nr	3, 6, 9 months	S. Liu, N. G. Xiao, et al. (2015)
2015	China	3–9 months	S. Liu, W. S. Xiao, et al. (2015)
2016	UK	11 months	Begus et al. (2016)
2016	Taiwan	4, 6, 9 months	Chien et al. (2016)
2016	nr	6, 9 months	Quinn et al. (2016)
2016	nr	9 months	Markant et al. (2016)
2016	Germany	3, 6, 9 months	Fassbender et al. (2016)
2017	nr	9 months	Liberman et al. (2017)
2017	nr	5, 10 months	Pickron et al. (2017)
2017	USA	6, 8 months	Ellis et al. (2017)
2017	nr	3, 6, 9 months	Thomas and Fassbender (2017)
2017	nr	4, 6, 8,10, 12 months	Montoya et al. (2017)
2017	nr	11 months	Singarajah et al. (2017)
2017	China	3–9 months	Xiao, Quinn, et al. (2018)
2017	nr	6, 9 months	Safar et al. (2017)
2017	nr	10–11.5 months	Singh et al. (2017)
2017	nr	3.5 months, 6 months	Heron‐Delaney et al. (2017)
2018	China	7	Xiao, Wu, et al. (2018)
2018	nr	4–6, 10–12 months	Minar and Lewkowicz (2018)
2018	Malaysia	3–4, 8–9 months	Tham et al. (2018)
2018	nr	3, 6, 9, 12 months	Xiao, Mukaida, et al. (2018)
2018	Germany	9 months	Krasotkina et al. (2018)
2018	Israel	14 months	Ferera et al. (2018)
2018	nr	16 months	Weatherhead and White (2018)
2018	nr	6 months	Holvoet et al. (2018)
2018	China	6, 9 months	S. Liu et al. (2018)
2019	nr	11 months	de Klerk et al. (2019)
2019	Canada	6, 11 months	May et al. (2019)
2019	Germany	3, 6, 9 months	Fassbender and Lohaus (2019)
2019	Singapore	18–20 months	Singh et al. (2019)
2019	Italy	5, 9 months	Timeo et al. (2019)
2019	USA	9	Kelsey et al. (2019)
2020	UK	6, 9, 12 months	Prunty et al. (2020)
2020	Spain	14 months	Colomer and Sebastian‐Galles (2020)
2020	nr	6, 12, 18 months	Aktar et al. (2020)
2020	USA	7–12 months	Hwang et al. (2020)
2020	Singapore	24 months	Singh et al. (2020)
2020	nr	6, 11 months	Keenan and Markant (2020)
2020	Japan	8–9 months	Ujiie et al. (2020)
2020	Italy	1–2, 3–4 years	Castelli and Carraro (2020)
2020	nr	3, 6 months	Quinn et al. (2020)
2021	Australia	10–12 months	L. Liu et al. (2021)
2021	Canada	24–26 months	Weatherhead and White (2021)
2021	Canada	4 months	Orena and Werker (2021)
2021	Canada	18 months	Weatherhead et al. (2021)
2021	USA	20–30 months	Qin (2021)
2021	Germany	6, 9 months	Krasotkina et al. (2021b)
2021	nr	18, 24 months	Laible et al. (2021)
2021	Japan	5–6, 8–9 months	Ujiie et al. (2021)
2021	California	20 months	Pronovost and Scott (2021)
2021	France	9, 12 months	de Boisferon et al. (2021)
2021	Israel	11 months	Ferera et al. (2021)
2021	USA	14 months	Haynes et al. (2021)
2021	Germany	12 months	Krasotkina et al. (2021a)
2021	California	7.5–10.5 months	Oakes et al. (2021)
2021	USA	6–7 months	Quinn et al. (2021)
2022	nr	10 months	Roth and Reynolds (2022)
2022	France	9, 12 months	Damon et al. (2022)
2022	Singapore	3, 6, 6–7, 9 months	Singh et al. (2022)
